# THE ERA OF CHATGPT: RECOMMENDATIONS FOR THE INTEGRATION OF LLMS IN GERONTOLOGY

**DOI:** 10.1093/geroni/igae098.1920

**Published:** 2024-12-31

**Authors:** Ari Friedman

**Affiliations:** University of Pennsylvania, Philadelphia, Pennsylvania, United States

## Abstract

Large language models (LLMs) and the platforms they support such as ChatGPT have experienced an exponential growth in popularity and use in the last year. ChatGPT surpassed100 million users in just two months after it was released to the public. These technological advances introduce opportunities for health care applications and it is anticipated that LLMs and various chatbot applications may significantly redesign clinical and health related processes and services. More specifically, in the space of gerontology, the use of Generative AI is anticipated to inform applications including automating clinical decision making, and even predicting cognitive decline based on end user data, providing digital companionship chatbots for isolated older adults, processing and increasing the accessibility of health education material and assisting in the documentation of care transitions. Rather than aiming to anticipate how companies will use LLMs to disrupt the field of gerontology, we have the opportunity and responsibility to define how ethical and appropriate gerontological care will shape the training of LLMs and the creation of guidelines for the use of Generative AI in gerontology. In December 2023 a group of national experts in the fields of gerontology, LLMs, AI and bioethics convened with the goal to propose guidelines to inform appropriate use of LLMs for systems that target older adults and persons with dementia and recommendations for the promotion of transparency in the design of LLMs for gerontology. This presentation will provide insights into these recommendations and guidelines for the role of LLMs in gerontology.

